# Effect of Na_3_PO_4_ on the Hydration Process of Alkali-Activated Blast Furnace Slag

**DOI:** 10.3390/ma9050395

**Published:** 2016-05-20

**Authors:** Lukáš Kalina, Vlastimil Bílek, Radoslav Novotný, Miroslava Mončeková, Jiří Másilko, Jan Koplík

**Affiliations:** Materials Research Centre, Faculty of Chemistry, Brno University of Technology, Brno 612 00, Czech Republic; bilek@fch.vut.cz (V.B.J.); xcnovotny2@fch.vut.cz (R.N.); xcmoncekova@fch.vut.cz (M.M.); masilko@fch.vut.cz (J.M.); koplik@fch.vut.cz (J.K.)

**Keywords:** alkali activated cement, granulated blast furnace slag, retardation

## Abstract

In recent years, the utilization of different non-traditional cements and composites has been increasing. Alkali-activated cementitious materials, especially those based on the alkali activation of blast furnace slag, have considerable potential for utilization in the building industry. However, alkali-slag cements exhibit very rapid setting times, which are too short in some circumstances, and these materials cannot be used for some applications. Therefore, it is necessary to find a suitable retarding admixture. It was shown that the sodium phosphate additive has a strong effect on the heat evolution during alkali activation and effectively retards the hydration reaction of alkali-activated blast furnace slag. The aim of the work is the suggestion of a reaction mechanism of retardation mainly based on Raman and X‑ray photoelectron spectroscopy.

## 1. Introduction

In recent years, a diverse selection of admixtures has been used to retard the setting in alkali-activated cements, although their activity varies substantially [1]. The research was initially focused on the retardants commonly used for Portland cement. Wu *et al.* [2] observed that potassium or sodium tartrate did not show any effect on the initial setting time, but slightly shortened the final setting time. The use of borates as retardants for Portland cement is also very well known. However, Nicholson *et al.* [3] reported that borates added to alkali-activated fly ash (class C) did not influence the setting behavior; conversely, the strength of the binders was negatively affected by a high amount of borates. Some admixtures used as setting accelerators in Portland cement systems have the opposite effect when used in alkali-activated materials. Brought *et al.* [4] investigated that the addition of NaCl significantly retarded both setting and strength development at high levels, but at low addition levels, *i.e.*, 4% or less by weight of slag, NaCl acted as an accelerator. Another possibility is the usage of phosphoric acid or its salts. Chang *et al.* [5] concluded that using solely phosphoric acid increased the setting time, but reduced the compressive strength at an early age. Gong and Yang [6] observed the strong retardant effect of sodium phosphate on alkali-activated red mud slag cementitious material. However, Shi and Li [7] found no retardation effect of Na_3_PO_4_ on alkali-activated phosphorus slag. From the published studies it is thus apparent that the nature and dosage of added admixtures and also the types of activated raw materials have a significant effect on the retardation of the setting process of the alkali-activated materials.

Unfortunately, the reaction mechanism of the retarding admixtures has not fully been explained [1]. Lee and Deventer [8] tried to suggest the hydration kinetics of PO_4_^3−^ in fly ash-based alkali-activated materials. They assumed that phosphate anions have strong affinities to Ca^2+^ cations. The value of the solubility product equilibrium constant for Ca(OH)_2_ is very low (p*K*_sp_ = 5.26); therefore, dissolved calcium cations from fly ash precipitated as Ca_10_(PO_4_)_6_(OH)_2_ (hydroxyapatite) instead of Ca(OH)_2_. On the other hand, Shi and Day indicate a different reaction mechanism [9] in alkali-activated blast furnace slag. From their study it seems that the formation of Ca_3_(PO_4_)_2_ retards the activation of slag as usually observed during the hydration of Portland cement. It is obvious that the final reaction products from the retardation process depend on used raw materials but the detailed action of phosphate in alkali-activated systems remains unclear.

The current study presents the possibilities of using sodium phosphate as an effective retardant for alkali-activated blast furnace slag-based materials and clearly explains the retarding effect which is described in more detail compared to the other published reaction mechanisms.

## 2. Materials and Methods

### 2.1. Materials and Sample Preparation

Blast furnace slag (BFS) obtained from ArcellorMittal Ostrava, Inc. (Ostrava, Czech Republic) ironworks was used as the raw material. The chemical composition of BFS determined by X-ray fluorescence spectroscopy (XRF) is given in Table 1. The phase composition of BFS measured by powder X‑ray diffraction (XRD) revealed the presence of merwinite, melilite, β-C_2_S and calcite. Water glass (molar ratio of SiO_2_/Na_2_O = 2.00) used as an alkali activator was produced by Vodní Sklo, Inc. (Brno, Czech Republic). Sodium phosphate (SP) additive was obtained from Sigma-Aldrich (St. Louis, MO, USA).

Alkali-activated samples were made by mixing water and water glass solution with the mixture of BFS and specific addition of sodium phosphate (recalculated to wt % of P_2_O_5_ contained in SP) for three minutes to produce homogenous pastes. Individual additions of SP were 0.5, 1.0, 2.5 and 5.0 wt % of P_2_O_5_ in sodium phosphate calculated on BFS content. The water glass solution and BFS ratio was set to 0.25 for all samples and the additional water was always mixed with water glass in the constant weight ratio 1:1. These pastes were cast into the polystyrene crucibles covered with teflon foil to prevent drying of the system. The specific quantity of the samples with the addition of SP was then taken in times defined by the microcalorimeter results, the hydration was then quenched by acetone and subsequently subjected to Raman and XPS spectroscopy. Due to the high amount of amorphous phase in the samples the usage of powder X‑ray diffraction as common silicate analytical technique was not satisfactory.

### 2.2. Physical‑Mechanical Tests

Initial setting time was determined according to the standard procedure ČSN EN 196-3 using Vicat´s device. Compressive strength tests were based on ČSN EN 196-1 and were carried out by means of compressive and bending strength on tester Desttest 3310 (Betonsystem Ltd., Brno, Czech Republic). The strengths were tested at the age of one, seven and 28 days. The workability of fresh mortars was measured using a flow table spread test (ČSN EN 1015-3). The diameter was measured in four directions after 15 blows with the jolting table. The final value was the arithmetic mean of these measurements.

### 2.3. Heat Evolution Rates Measurement

The evolution of hydration heat was monitored by means of TAM Air isothermal calorimeter (TA instruments, Wetzlar, Germany). The test samples of BFS and sodium phosphate were weighted and uniformly distributed to the glass-closed ampules (15 mL). The solution of water and alkali activator was weighted in a syringe. The water ratio was the same as in the preparation process of pastes and the mass of slag was 4 g. The measurements of heat evolution were performed at constant temperature of 25 °C ± 0.02 °C. The samples with BFS and SP as well as the syringe with the activator and water were heated to testing temperature prior to mixing in the admix ampule directly in the calorimeter. When the thermal equilibrium was achieved, BFS with SP and alkali activator were mixed by injecting the solution into the ampule. The time of mixing was three minutes. The heat evolution was recorded as the heat flow (mW/g).

### 2.4. Raman Spectroscopy

Raman scattering measurements were performed using Nanofinder S micro-Raman spectrometer (SOL instruments, Minsk, Belarus) equipped with a confocal microscope (Nikon, Amsterdam, The Netherlands). The Raman scattering spectra were excited by 633 nm with 10 mW power. The system was calibrated on silicon (518.2 cm^−1^). The beam was focused on the samples with a 20× microscope objective with a numerical aperture of 0.45. The exposure time was 100 s and 600 grating with 2.7 cm^−1^ resolution. All measurements were performed at room temperature in ambient atmosphere.

### 2.5. X-ray Photoelectron Spectroscopy

X-ray photoelectron spectroscopy (XPS) were carried out with Axis Ultra DLD spectrometer (Kratos Analytical Ltd., Manchester, UK) using a monochromatic Al Kα (hν = 1486.7 eV) X-ray source operating at 150 W (10 mA, 15 kV). The spectra were obtained using an analysis area of ~300 × 700 µm. The Kratos charge neutralizer system (Kratos Analytical Ltd., Manchester, UK) was used for all analyses. High resolution spectra were measured with the step size 0.1 eV and 20 eV pass energy. Instrument base pressure was 2·10^−8^ Pa. The spectra were analyzed using CasaXPS software (version 2.3.15) and charge corrected to the main line of the carbon C 1s spectral component (C–C, C–H) set to 284.80 eV. A standard Shirley background was used for all sample spectra.

## 3. Results and Discussion

### 3.1. Physical‑Mechanical Properties

**Figure 1:**
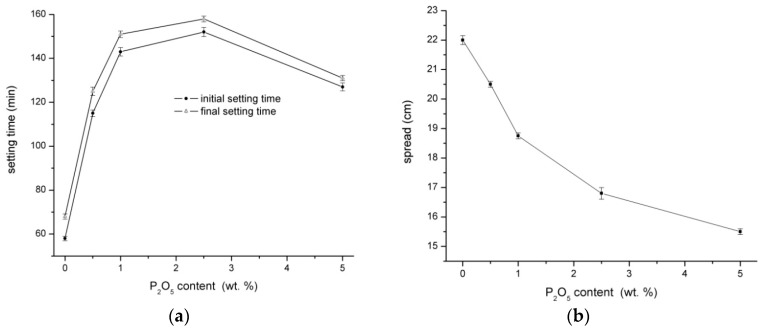
Influence of Na_3_PO_4_ on the initial and final setting time (**a**) and workability (**b**) of alkali-activated BFS.

The influence of different additions of sodium phosphate is shown in Figure 1. Both initial and final setting time were prolonged with the sodium phosphate content but only to a certain limit of P_2_O_5_ content (Figure 1a). The maximum of retardation is observed for the content of 2.5 wt % of P_2_O_5_; above that value, the time of setting is shortened, and therefore this content was chosen for further study of the reaction mechanism via Raman and XPS analyses. In general, the addition of SP considerably delays the initial time of setting which has been already verified in previous studies [5,6,7,8].

The workability determination of the flow table spread test is shown in Figure 1b. The workability gradually decreased with the higher content of SP. With respect to the initial and final setting time measurement, the reason of the workability decline is not related to the formation of the binder phase but is caused by the loss of mixing water which is very quickly bonded to the created phosphate hydrates, as verified by Raman spectroscopy.

**Figure 2:**
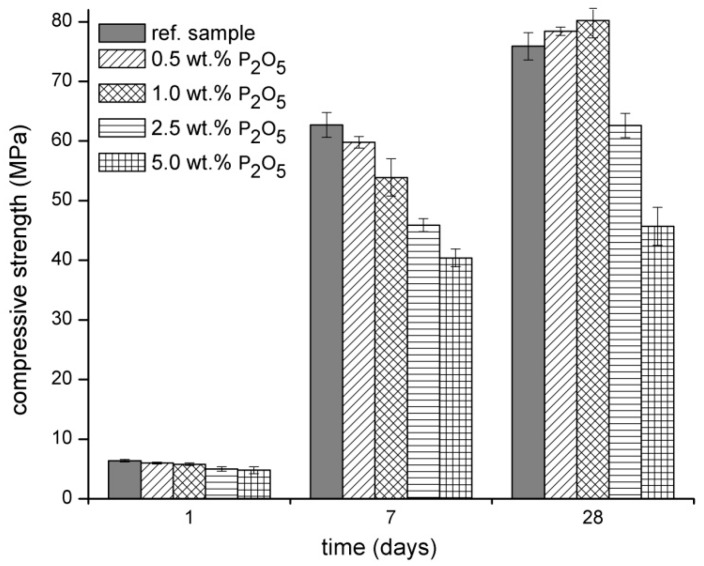
Influence of Na_3_PO_4_ on the compressive strength development of alkali-activated BFS.

**Figure 3:**
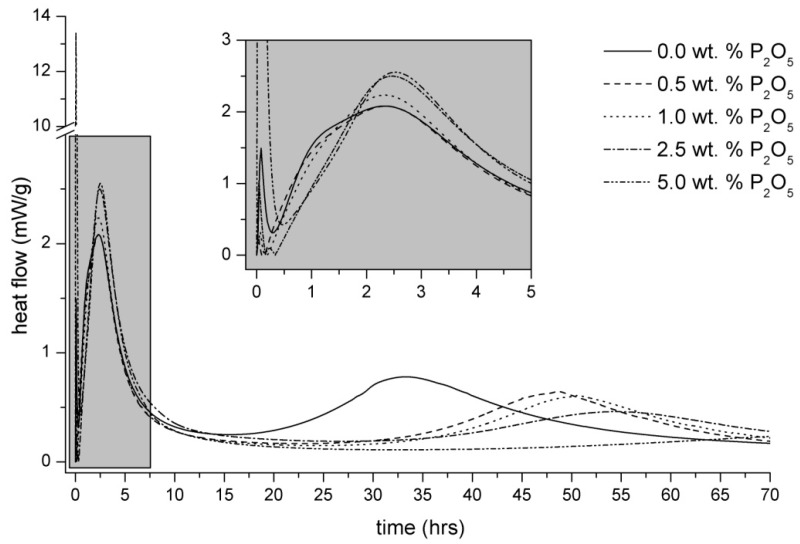
Influence of Na_3_PO_4_ on the initial setting of alkali-activated BFS.

The compressive strength development is shown in the Figure 2. The early strengths of samples with the addition of SP are slightly lower compared to the reference sample. This effect is probably connected to the secondary formation of CSH gel. The formation of secondary CSH gel in the reference sample starts to increase earlier, which positively affects the mechanical properties after one day (Figure 3). After seven days, the obvious trend of lower compressive strength with the greater addition of SP is observed. The addition of SP decreases the secondary formation of CSH gel which is well correlated with the heat evolution in Figure 3. However, after 28 days, the samples with a lower addition of SP (0.5 and 1.0 wt %) showed higher compressive strength compared to the reference sample. This beneficial strength development could play an important role in the production of alkali-activated materials, because the lower SP content (up to 1.0 wt %) significantly retards the initial and final setting time, and it also slightly worsens the workability but improves the final mechanical properties.

### 3.2. Heat Evolution

The hydration heat evolution of alkali activated BFS without or with the specific addition of P_2_O_5_ contained in SP is shown in Figure 3. The first peak is associated with wetting and dissolution of slag and phosphates particles as well as with the formation of sodium phosphate hydrates. The second peak is mainly connected with the formation of primary CSH gel through the reaction of SiO_4_^4−^ from water glass and Ca^2+^ dissolved from the surface of slag grains. In this hydration period, the effect of the added sodium phosphate is also reflected. The total heat evolution values corresponding to the second peak increase with the greater addition of SP, which can be attributed to the formation of new hydrogen phosphate phases as discussed further. Finally, the third peak belongs to the secondary formation of CSH gel. It is evident that the hydration reactions start earlier in pure alkali-activated BFS systems than in the case of alkali-activated slag with a retardant admixture. The calorimetry results clearly show that the heat evolution changes with the addition of sodium phosphates. Unfortunately, it is not possible to explain the reaction mechanism only from thermocalorimetric curves because the released heats connected with the formation of different phases (CSH gel, calcium phosphates and phosphate hydrates) are overlapped. Therefore, the employment of suitable testing methods, such as the combination of Raman spectroscopy and X-ray photoelectron spectroscopy, is needed. Due to the high amount of amorphous phase in the samples, these methods are most appropriate for the study of the reaction mechanism of the phosphate additive in alkali-activated binders.

### 3.3. Raman Spectroscopy

**Figure 4:**
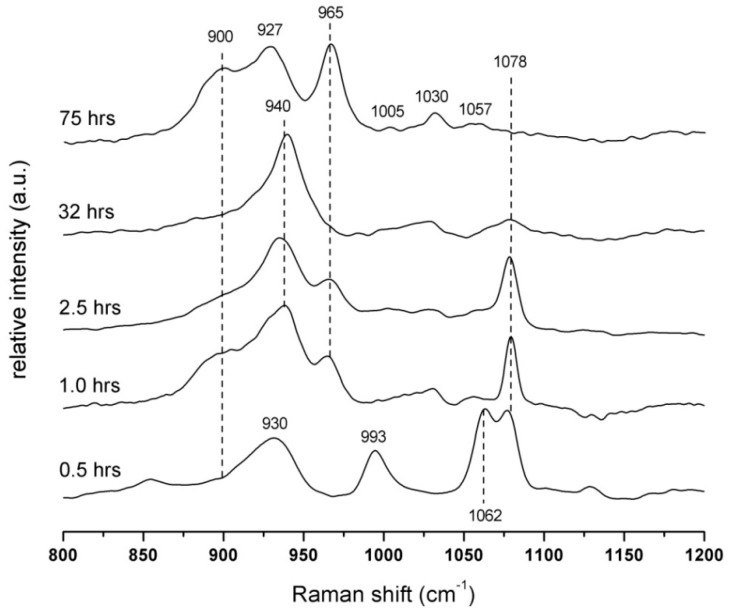
Raman spectra of alkali-activated BFS with Na_3_PO_4_ recalculated to the 2.5 wt % addition of P_2_O_5_.

The Raman investigations show five spectra at various times of alkali activation (Figure 4). The first spectrum, 30 min after mixing, reveals the characteristic band at 930 cm^−1^ corresponding to the symmetric stretching vibration υ_1_(A_1_) of tetrahedral PO_4_^3−^ [10,11]. All spectra show the presence of Na_3_PO_4_ hydrates. This hydration process of Na_3_PO_4_·xH_2_O is connected with the decrease in the asymmetric stretching mode around 1078 cm^−1^ υ_3_(F_2_), which presents the distortion of symmetry of PO_4_^3−^ in the crystal structure. The shoulder at ~900 cm^−1^ indicates the changes in the symmetry site of the PO_4_^3−^ characteristic of the hydrated form of Na_3_PO_4_·7H_2_O [12]. After 0.5 h from the beginning of the hydration process, two bands at 993 and 1062 cm^−1^ appear and they are attributed to the P–O symmetric stretching vibration υ_1_(A_1_) and both P–O, P–OH asymmetric stretching vibrations υ_3_(F_2_) in the H_2_PO_4_^−^ unit, respectively [13]. After one hour, the hydration process of Na_3_PO_4_·xH_2_O continues, and it is connected with the shift of the band position from 930 to 940 cm^−1^ [12]. As could be seen from the spectra, the phase with the H_2_PO_4_^−^ unit completely disappears and the new band at 965 cm^−1^ of the symmetric stretching vibration of υ_1_(A_1_), typical for HOPO_3_^2−^, is observed [13]. This band does not remain in the system for long and it completely diminishes over time at around 32 h. However, after 75 h, the band at 965 cm^−1^ arises again. In this case, the very strong band is typical for the calcium hydroxyapatite Ca_x_(PO_4_)_y_(OH)_z_ structure which also corresponds to the growth of the weak bands at 1057, 1030 and 1005 cm^−1^ assigned to the asymmetric stretching modes υ_3_(F_2_) of the P–O bond in the tetrahedral PO_4_^3−^ group [14,15]. The suggested mechanism of created phosphate phases is further discussed in XPS measurements.

### 3.4. X-ray Photoelectron Spectroscopy

**Figure 5:**
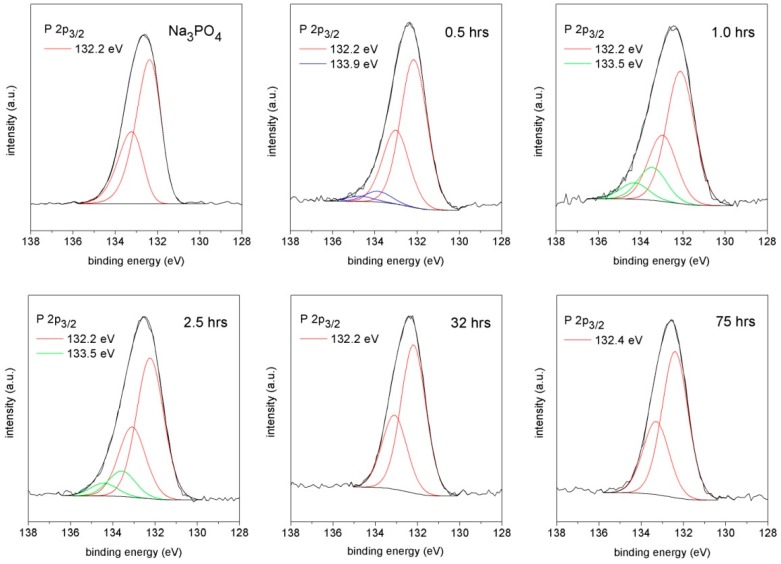
XPS spectra of alkali-activated BFS with Na_3_PO_4_ recalculated to the 2.5 wt % addition of P_2_O_5_. (The energy separations for the P 2p_3/2_ and P 2p_1/2_ lines were fixed at 0.80 eV.)

The core level P 2p_3/2_ positions of samples subjected to measurements are summarized in Table 2 and provide an insight into the binding of the phosphate in the system. The high resolution spectra of samples quenched at the same times as in the case of the Raman spectroscopy are presented in Figure 5.

The first P 2p spectrum represents a pure Na_3_PO_4_ compound with a typical binding energy (BE) of PO_4_^3−^ equal to 132.2 eV. Strong peaks with this BE appear in each spectrum, which indicates the presence of the Na_3_PO_4_ phase at any time during the alkali activation. However, other components representing different chemical states of phosphorus are detected. According to the previous experimental measurements, the phosphate anions of the [PO_n_(OH)_m_]^y−^ type generally follow a systematic rule. The contribution of covalently bonded OH ligands (*m*) to “free” O ligands (*n*) increases together with the binding energy of P 2p. The stepwise increase from PO_4_^3−^ up to H_3_PO_4_ is typically about 1 eV in every OH ligand presented in the structure [16,17]. Therefore, the P 2p_3/2_ component with BE at 133.9 eV in 30 minutes of alkali activation belongs to the H_2_PO_4_^−^ units verified with Raman spectroscopy as well (993 and 1062 cm^−1^ bands). The P 2p spectra t 1.0 and 2.5 h indicate the increase of BE to 133.5 eV, which corresponds to phosphate with only one hydroxyl unit. The results of Raman spectroscopy together with the XPS investigations revealed the existence of HOPO_3_^2−^. XPS measurements also correspond to the Raman spectroscopy concerning the disappearance of this phase 32 h after mixing, which comes just before the secondary formation of the CSH gel (Figure 3). The calcium ions bonded so far in the hydrogen phosphate structure likely move into the solution and incorporate the created phases with the always higher value of the negative logarithms of the solubility product equilibrium constant pK_sp_ such as secondary CSH gel (pK_sp_ = 8.16 for CSH gel with low C/S ratio [18]) and calcium hydroxyapatites (pK_sp_ = 115.50 for Ca_10_(PO_4_)_6_(OH)_2_ [19]). The P 2p_3/2_ component in the spectrum of 75 h with BE at 132.4 eV does not show any hydrogen phosphate forms, which is also in good agreement with the Raman investigations. The shift in the BE compared to the pure Na_3_PO_4_ P 2p spectrum could be attributed to sodium phosphates with a small addition of stable calcium hydroxyapatite of which the binding energy is slightly higher [16,20]. The peak positions of both phases are considerably overlapped and should not be deconvoluted.

## 4. Conclusions

The results from Raman and XPS analyses combined with microcalorimetry measurements were beneficially used to clarify the mechanism of retardation in alkali-activated materials with the addition of sodium phosphate. It was assessed that Ca^2+^ ions formed after the dissolution of alkali-activated blast furnace slag in a highly alkaline environment bond to the phosphate anion from the retardant agent (Na_3_PO_4_). The formation of calcium dihydrogen, later hydrogen phosphate structures, arises. The deficiency of calcium ions in the solution causes the nucleation and the growth of the CSH phase to be poisoned and the initial setting time is prolonged. Their formation, nevertheless, also strongly affects the delayed formation of secondary CSH gel compared to the system without the retarding agent, which may affect the mechanical properties of alkali-activated materials at an early age. The stability of the created calcium hydrogen phosphate phase in the system is temporary, which leads to its dissolution and the formation of less soluble phases such as secondary CSH gel and calcium hydroxyapatite.

## Figures and Tables

**Table 1:** Chemical composition of used BFS as determined by XRF.

Raw Material	Chemical Composition wt %									
BFS	SiO_2_	Al_2_O_3_	CaO	Na_2_O	K_2_O	MgO	SO_3_	Fe_2_O_3_	TiO_2_	MnO
	34.7	9.1	41.1	0.4	0.9	10.5	1.4	0.3	1.0	0.6

**Table 2:** The binding energies and peak widths (FWHM) of P 2p_3/2_ components.

Component	Binding Energy (FWHM)/eV		
	Na_3_PO_4_	0.5 h	1.0 h
P 2p_3/2_	132.2 (1.4)	132.2 (1.4)	132.2 (1.4)
		133.9 (1.4)	133.5 (1.4)
	**2.5 h**	**32 h**	**75 h**
	132.2 (1.4)	132.2 (1.4)	132.4 (1.4)
	133.5 (1.4)		

## Abbreviations

The following abbreviations are used in this manuscript:

*K*_sp_	solubility product constant
BFS	blast furnace slag
XRF	X-ray fluorescence
XRD	X-ray diffraction
β-C_2_S	β-dicalcium silicate
SP	sodium phosphate
XPS	X-ray photoelectron spectroscopy
CSH gel	calcium-silicate-hydrate gel
FWHM	full width at half maximum
BE	binding energy
υ_1_(A_1_)	symmetric stretching vibration
υ_3_(F_2_)	asymmetric stretching vibration
